# The weak correlation between serum vitamin levels and chronic kidney disease in hospitalized patients: a cross-sectional study

**DOI:** 10.1186/s12882-021-02498-5

**Published:** 2021-08-26

**Authors:** Yong Wang, Ying Zheng, Pu Chen, Shuang Liang, Pengfei He, Xiaolei Shao, Guangyan Cai, Xiangmei Chen

**Affiliations:** Department of Nephrology, State Key Laboratory of Kidney Diseases, First Medical Center of Chinese People’s Liberation Army General Hospital, Chinese People’s Liberation Army Institute of Nephrology, National Clinical Research Center for Kidney Diseases, Fuxing Road 28, 100853 Beijing, China

**Keywords:** Chronic kidney disease, Glomerular filtration rate, Vitamin, Deficiency, Cross-sectional study

## Abstract

**Background:**

Chronic kidney disease (CKD) has become a global public health problem. Accumulating evidence suggested that vitamins play important roles in the progression of CKD.

**Methods:**

A cross-sectional study was conducted to investigate the vitamin status of patients with CKD at stage 1–5. The serum concentrations of 9 vitamins, vitamin A, B1, B2, B6, B9, B12, C, D, and E were measured by electroanalytical method with a Multi-Vitamin Analyzer. Pearson correlation and multiple linear regression between serum level of vitamins were analyzed.

**Results:**

The median levels of vitamin A, B1, B2, B6, B9, B12, C and E were within the reference ranges or on the borderline. Vitamin D deficiency was found in all patients. Weak correlation was found between vitamin A or vitamin D and estimated glomerular filtration rate (eGFR). The Pearson correlation coefficient were − 0.21766 and 0.19752, respectively. Hypertension, diabetes mellitus, and atherosclerosis were the major comorbidities.

**Conclusions:**

For the first time, the serum levels of 9 vitamins were measured simultaneously in patients with CKD at different stages. Vitamin D deficiency was found in all patients. Weak correlation between vitamin A or vitamin D and eGFR was found.

## Background

Chronic kidney disease (CKD) refers to kidney damage and decreased renal function, which can be classified into 5 stages according to KDOQI guidelines [1]. The global prevalence of CKD (stage 1–5) was estimated as 13.4 % with a 95 % confidence interval (CI) from 11.7 to 15.1 %. The prevalence of stages 3 to stage 5 was 10.6 % (95 %CI: 9.2-12.2 %) [2]. In China, about 119 million people are suffering from CKD [3]. Individuals with CKD are facing an increased risk for kidney failure, cardiovascular disease, and mortality despite standard interventions for managing the conventional risk factors associated with it [4]. CKD has been recognized as one of the most serious health problems worldwide, increasing the global burden of morbidity and mortality, as well as consuming scarce health resources. Therefore, deeper understanding of its mechanism and new intervention approaches based on non-traditional risk factors are of great importance [3–6].

Over the years, both basic research and clinical trials have improved our understanding of the interaction between disordered vitamins and CKD [7, 8]. CKD has been identified as a risk factor for vitamin D deficiency, which is highly prevalent in patients with CKD, especially those with end-stage renal disease (ESRD) and kidney transplant recipients [9]. The deficiency of serum 25(OH)D3 (the major circulating form of vitamin D) is present in all subsequent stages of CKD [10], and the prevalence increases as kidney function declines [11]. Individuals with 25(OH)D3 levels lower than 37.5 nM (15 ng/mL) had a higher risk for all-cause mortality despite adjustments for CKD stage and confounding factors [12]. To date, no randomized clinical trial (RCT) aiming to verify the beneficial effect of 25(OH)D3 supplementation on the outcomes of CKD has been identified [13]. The correlation between vitamin D and the prognosis of CKD needs further verification.

Renal dysfunction of CKD patients is reportedly associated with a decrease in plasma vitamin C level. Moreover, decreased vitamin C may cause endothelial dysfunction via an increase in oxidative stress in non-diabetic chronic kidney disease patients [14]. Hyperhomocysteinemia (hHcy) is recognized as a novel risk factor that contributes to the increased incidence of cardiac event among CKD patients. hHcy occurs in about 85 % of chronic kidney disease (CKD) patients, mainly because of the deficiency of vitamin В6, В9 and В12 [15]. It is therefore reasonable to assume that lowering homocysteine by B vitamin supplementation would finally lead to a reduction of cardiovascular complication and mortality. However, interventional studies through B vitamin supplementation to reduce homocysteine level did not show favorable clinical outcomes [16]. Despite this, folic acid (vitamin B9) treatment was associated with a significant reduction in the odds of CKD progression among mild to moderate CKD patients. A large RCT in China showed statistically significant reductions in the risk of first stroke and CKD progression with the addition of folic acid to Enalapril in adults with hypertension [17]. Recently, the genetic polymorphisms in vitamin metabolism have received great attention, suggesting that the evaluation of vitamin status from different gene polymorphisms may not be comparable [18]. Taken together, it is of great importance to study the pathophysiological role of the vitamins and discuss their correlation with CKD progression under different genetic background. This study aimed to investigate the vitamin status of patients with CKD in China and evaluate the correlation between serum vitamin level and progression of renal function.

## Methods

### Study design

CKD was defined as abnormalities of kidney structure or function, present for more than 3 months, with implications for health (Not graded). The diagnostic thresholds were estimated glomerular filtration rate (eGFR) of less than 60 mL/min per 1.73 m^2^ and an albumin-creatinine ratio (ACR) of 30 mg/g or greater were retained [19, 20] CKD was classified into five stages per the KDOQI guidelines using thresholds of estimated glomerular filtration rate (eGFR) within the CKD range and/or evidence of structural renal changes [21]. The eGFR value for different CKD stages were: eGFR ≥ 90 (stage 1), 60 ≤ eGFR < 90 (stage 2); 30 ≤ eGFR < 60 (stage 3); 15 ≤ eGFR < 30 (stage 4); and eGFR < 15 (stage 5).

From May 2018 to September 2019, consecutive patients with primary diagnosis of CKD and hospitalized at the Department of Nephrology, the First Medical Center of the General Hospital of People’s Liberation Army, were invited to participate the study. Exclusion criteria were (1) acute kidney injury (any of the following: increase in serum creatinine (sCr) by ≥ 0.3 mg/dl (≥ 26.5 µmol/l) within 48 h; or an increase in serum creatinine to ≥ 1.5 times baseline for 7 days; or a urine volume < 0.5 ml/kg/h for 6 h.); [22] (2) younger than 18 years old; (3) have participated in other kidney disease-pertinent clinical trial; (4) history of organ or bone marrow transplantation; (5) missed important analytic variables.

The study was approved by ethics committee of First Medical Center of Chinese People’s Liberation Army General Hospital and carried out in accordance with the declaration of Helsinki.

### Anthropometric data collection

Following our standardized protocols, demographic data and anthropometric measurements were collected by trained investigators. eGFR was calculated by the CKD Epidemiology Collaboration (CKD-EPI) equation [23]. Body mass index (BMI) was calculated as body weight (kg)/squared height (m^2^). Complete blood count, alkaline phosphatase (ALP), calcium, phosphorus, iron, albumin, creatinine, cystatin C, total cholesterol (CHOL), triglyceride (TG), high‑density lipoprotein (HDL), low‑density lipoprotein (LDL), homocysteine (Hcy) and other biochemical and clinical tests were performed as routine investigations. Renal biopsy was performed to investigate the possible etiology of CKD.

### The analysis of serum vitamins

Serum concentrations of 9 vitamins, vitamin A, B1, B2, B6, B9, B12, C, D and E were measured by electroanalytical method with Multi-Vitamin Analyzer (LK3000V, Tianjin Lanbiao Electronic Technology Development Co. Ltd, China) following the manufacturer’s instructions. Briefly, 10ml venous blood sample was collected after 12-hour fasting and was centrifuged at 3000 rpm for 10 min quickly after collection. The separated serum sample were stored at 4 °C for testing. Vitamin E had to be tested within 24 h of sample collection, while other vitamins could be tested within 3 days. Sample preparation solution and vitamin standard solution for calibration were provided by the Tianjin Lanbiao Electronic Technology Development Co. Ltd, China. The detection limitation for different vitamins were vitamin A: 8.5 × 10^− 10^ mol/L, vitamin B1: 3.4 × 10^− 9^ mol/L, vitamin B2: 4.3 × 10^− 8^ mol/L, vitamin B6: 1.5 × 10^− 10^ mol/L, vitamin B9: 2.3 × 10^− 10^ mol/L, vitamin B12: 1.7 × 10^− 12^ mol/L, vitamin C: 2.8 × 10^− 6^ mol/L, vitamin D: 3.8 × 10^− 9^ mol/L, vitamin E: 6.0 × 10^− 6^ mol/L.

The concentration of a specific vitamin in serum sample below the following reference value was defined as deficiency, vitamin A: 0.70 µM, vitamin B1: 80.3 nM, vitamin B2: 106 nM, vitamin B6: 20 nM, vitamin B9: 10 nM, vitamin B12: 150 pM, vitamin C: 11 µM, vitamin D: 50 nM, and vitamin E: 23 µM. Normal value of serum homocysteine is below 15 µM [24–31].

### Statistical analyses

All statistical analyses were performed using SAS (version 9.3; Cary, NC). Each variable was tested for normality before statistical analysis. Data were presented as median (interquartile range, IQR) or frequency (%). The difference between two groups were analyzed by the chi-squared test, Fisher’s Exact test, Kruskal-Wallis test or Student-Newman-Keuls test where appropriate. Pearson correlation coefficient was calculated to characterize the correlation between two variables. Multiple linear regression analysis was performed to examine the correlation between significant variables and eGFR. All *p*-values were two-tailed and those less than 0.05 were considered statistically significant.

## Results

In total, 759 CKD patients were included in this study. Based on their eGFR values, the participants were assigned to 5 different stages according to the classification criteria described in the Methods section. The demographic and clinical characteristics of the cohort by different CKD stages were presented in Table 1. About three quarters of the patients were from stage 1 to stage 3, while the rest were from stage 4 and stage 5 equally. Patients in advanced stages were older than those in the lower stages (p < 0.001). More female patients were found in stage 1, whilst more males were found in other stages (p < 0.001). No statistically significant difference was found in the body mass index (BMI) of different stages. Serum concentrations of prealbumin, cystatin-C, alkaline phosphatase (ALP), creatinine, Hcy, urine protein, blood urea nitrogen (BUN), and uric acid increased along with the severity of CKD. The Hcy of patients with CKD at stage 1 were within the normal range, though it started increasing from stage 2 until stage 5, when Hcy levels were 2.58 times that of stage 1. Other indicators, such as hemoglobin (Hb), urine osmolality, and total iron binding capacity (TIBC) followed a decreasing trend in relation to the increasing severity of CKD.

**Table 1:** Demographic and Clinical characteristics of CKD patients

Variable	CKD					*p*-value
	Stage 1 (*n* = 179)	Stage 2 (*n *= 187)	Stage 3 (*n* = 201)	Stage 4 (*n* = 97)	Stage 5 (*n* = 95)	
Age (year)	41 (30, 49)	45 (33, 55)	48 (36, 59)	51 (40, 62)	50 (36, 60)	0.000
Sex (M/F)	57/122	124/62	148/53	70/27	69/26	0.000
BMI (kg/m^2^)	25.14 (22.04, 27.61)	25.39 (23.46, 28.06)	25.68 (22.84, 28.59)	24.52 (23.13, 26.71)	25.32 (23.05, 27.99)	0.166
Ca (mM)	2.16 (2.08, 2.25)	2.20 (2.09, 2.29)	2.19 (2.08, 2.30)	2.20 (2.11, 2.26)	2.11 (1.95, 2.19)	0.000
Fe (mM)	14.00 (9.60, 19.30)	15.90 (12.55, 20.05)	14.40 (11.00, 18.35)	12.60 (9.70, 16.20)	9.75 (6.90, 14.20)	0.000
P (mM)	1.21 (1.09, 1.33)	1.19 (1.05, 1.35)	1.21 (1.07, 1.37)	1.26 (1.12, 1.43)	1.85 (1.56, 2.20)	0.000
WBC (10^9^/L)	6.72 (5.47, 8.26)	6.48 (5.57, 8.05)	6.65 (5.38, 8.0)	6.57 (5.37, 7.7)	6.78 (5.63, 8.43)	0.834
Hb (g/L)	128 (116, 140)	138 (124, 150)	128 (115, 146)	114 (102, 127)	89 (80, 101)	0.000
CRP (mg/L)	0.9 (0.5, 1.0)	0.9 (0.6, 1.0)	0.9 (0.6, 1.1)	0.9 (0.6, 1.8)	1.0 (0.7, 2.7)	0.003
Prealbumin (mg/L)	272.0 (229.5, 338.0)	299.0 (251.0, 363.0)	307.5 (250.0, 371.0)	333.0 (264.0, 394.0)	336.0 (288.5, 431.0)	0.000
Urine protein (g/d)	1.23 (0.40, 2.89)	1.17 (0.49, 3.15)	1.50 (0.40, 3.18)	1.84 (0.79, 3.50)	2.80 (1.60, 4.34)	0.000
Albumin (g/L)	35.3 (30.3, 40.6)	36.6 (29.7, 41.3)	38.0 (32.2, 42)	38.8 (29.7, 41.4)	37.0 (33.0, 40.9)	0.084
Creatinine (µM)	63.4 (56.7, 70.8)	86.0 (80.0, 92.5)	125.7 (112.5, 146.5)	218.4 (190.8, 252.7)	615.3 (443.4, 851.5)	0.000
BUN (nM)	4.31 (3.59, 5.19)	5.34 (4.56, 6.14)	7.43 (6.11, 9.12)	11.33 (9.72, 14.38)	22.58 (17.85, 28.93)	0.000
Uric acid (µM)	326.2 (269.9, 376.0)	372.8 (324.4, 437.2)	397.1 (335, 470.2)	401.4 (336.3, 474.5)	434.9 (349.2, 516)	0.000
ALP (U/L)	53.5 (43.9, 62.9)	55.7 (45.2, 69.4)	60.4 (46.4, 73.8)	63.4 (50.2, 75.2)	67.0 (51.4, 82.0)	0.000
TC (mM)	4.53 (3.82, 5.63)	4.40 (3.71, 5.62)	4.18 (3.61, 5.28)	3.85 (3.32, 4.69)	3.96 (3.24, 5.01)	0.000
TG (mM)	1.67 (1.18, 2.55)	1.8 (1.26, 2.46)	1.85 (1.23, 2.83)	1.75 (1.26, 2.69)	1.82 (1.26, 2.33)	0.779
HDLC (mM)	1.12 (0.98, 1.43)	1.10 (0.88, 1.36)	1.02 (0.85, 1.29)	0.96 (0.80, 1.26)	0.94 (0.81, 1.14)	0.000
LDLC (mM)	2.91 (2.26, 3.79)	2.75 (2.22, 3.53)	2.62 (1.99, 3.67)	2.17 (1.72, 3.10)	2.32 (1.70, 3.00)	0.000
Hcy (µM)	10.7 (8.6, 13.4)	13.9 (11.8, 17.7)	18.6 (15.4, 24.2)	23.3 (17.2, 31.5)	27.6 (19.6, 36.9)	0.000
Cystatin-C (mg/L)	0.86 (0.74, 0.95)	1.04 (0.91, 1.17)	1.58 (1.33, 1.86)	2.47 (2.21, 2.94)	4.43 (3.79, 5.36)	0.000
TIBC (µM)	46.8 (40.7, 52.6)	44.9 (39.7, 50.6)	44.0 (36.8, 49.5)	40.8 (36.3, 46.3)	40.6 (34.7, 47.5)	0.000
HbA1c (%)	5.50 (5.20, 6.05)	5.50 (5.20, 5.80)	5.85 (5.25, 6.50)	6.10 (5.40, 6.80)	5.50 (5.20, 6.20)	0.008
IgA (mg/dl)	227 (177, 325)	239 (188, 338)	247 (188, 321)	219 (170, 300)	220 (168, 285)	0.206
IgM (mg/dl)	97.9 (67.8, 132.0)	84.9 (62.5, 121.0)	90.5 (62.8, 118.0)	92.4 (54.5, 133.0)	68.4 (48.8, 96.3)	0.002
IgG (mg/dl)	931 (684, 1114)	933 (661, 1190)	997 (779, 1250)	1040 (783, 1290)	995 (748, 1220)	0.029
C3 (mg/dl)	109.0 (92.7, 122.0)	108.0 (93.8, 120.0)	102.0 (86.9, 116.0)	94.4 (81.7, 111.0)	88.5 (71.2, 106.0)	0.000
C4 (mg/dl)	23.4 (18.8, 29.4)	24.7 (20.6, 29.2)	26.0 (21.5, 30.7)	26.1 (21.9, 33.6)	26.7 (22.4, 32.0)	0.004

Note: results were from blood samples unless otherwise specified; data were expressed as median (IQR); *BMI* body mass index; *WBC* white blood cell; *Hb* hemoglobin; *CRP* C-reactive protein; *BUN* blood urea nitrogen; *ALP* alkaline phosphatase; *TC* total cholesterol; *TG* triglyceride; *HDLC* high-density lipoprotein cholesterol; *LDLC* low-density lipoprotein cholesterol; *Hcy* homocysteine; *TIBC* total iron binding capacity; *HbA1c* glycated hemoglobin; *C3* complement component 3; *C4* complement component 4

Based on the results of renal biopsy, the pathological classification of CKD patients was displayed in Table 2. The pathological subgroups of CKD were not distributed equally in different stages (*p* < 0.001). The major pathological type of CKD in this cohort was IgA nephropathy (40.6 %) followed by membranous nephropathy at a prevalence of 31.5 %.

**Table 2:** Pathological diagnosis of CKD patients

Pathological subgroup	CKD					*p*-value
	Stage 1	Stage 2	Stage 3	Stage 4	Stage 5	
IgAN	37 (17.05)	62 (28.57)	80 (36.87)	26 (11.98)	12 (5.53)	0.000
MN	71 (42.26)	64 (38.09)	26 (15.48)	6 (3.57)	1 (0.60)	----
FSGS	1 (2.86)	5 (14.28)	23 (65.72)	4 (11.43)	2 (5.71)	----
OPN	16 (36.36)	12 (27.27)	9 (20.45)	3 (6.81)	4 (9.09)	----
SN	17 (24.29)	13 (18.57)	23 (32.86)	11 (15.71)	6 (8.57)	----

Note: data were expressed as frequency (%); *MN* membranous nephropathy; *IgAN* IgA nephropathy; *FSGS* focal segmental glomerulosclerosis; *OPN* other primary nephropathy; *SN* secondary nephropathy

Hypertension was the most common comorbidity in this cohort. 437 (75.47 %) patients were suffering from hypertension. Its incidence increased along with the increase of CKD severity, ranging from 29 % of stage 1 patients to 87 % of stage 5 patients. 140 (18.44 %) and 81 (10.67 %) patients had diabetes mellitus (DM) and atherosclerosis, respectively. The incidence rates of hypertension, DM, and atherosclerosis were higher in advanced stages than in lower stages (*p* < 0.001). The incidence rates of other comorbidities, such as liver disease (14.23 %), gastrointestinal diseases (11.86 %), respiratory diseases (10.93 %), cancer (5.80 %), and coronary heart disease (5.40 %) were relatively low, and no difference was found in different stages. More details of the common comorbidities in this cohort were shown in Table 3.

**Table 3:** Common comorbidities in CKD patients at different stages

Comorbidity	CKD					*p*-value
	Stage 1 (*n* = 179)	Stage 2 (*n* = 187)	Stage 3 (*n* = 201)	Stage 4 (*n* = 97)	Stage 5 (*n* = 95)	
Hypertension	52 (29.05)	86 (45.99)	136 (67.66)	80 (82.47)	83 (87.37)	0.000
Diabetes mellitus	19 (10.61)	15 (8.02)	43 (21.39)	30 (30.93)	33 (34.74)	0.000
Atherosclerosis	9 (5.03)	20 (10.70)	16 (7.96)	20 (20.62)	16 (16.84)	0.000
Liver disease	17 (9.50)	32 (17.11)	37 (18.41)	11 (11.34)	11 (11.58)	0.071
Gastrointestinal diseases	17 (9.50)	20 (10.70)	27 (13.43)	12 (12.37)	14 (14.74)	0.653
Respiratory diseases	26 (14.53)	19 (10.16)	24 (11.94)	6 (6.19)	8 (8.42)	0.235
Cancer	13 (7.26)	11 (5.88)	5 (2.49)	10 (10.31)	5 (5.26)	0.078
Coronary heart disease	6 (3.35)	7 (3.74)	8 (3.98)	11 (11.34)	9 (9.47)	0.011

Note: data were expressed as frequency (%)

The serum level of 9 vitamins were exhibited in Table 4. Most of the patients had their serum vitamin concentration above the reference levels. Few of them had their vitamin A levels on the borderline or below the reference value. It is worth noting that vitamin D deficiency was found in all of the participants. The concentration of vitamin D started decreasing from stage 3, and its level in stage 5 was lower than in stage 4, the concentration in the latter was lower than in stage 3 (*p* < 0.001). The changing of vitamin D level was associated with the severity of eGFR staging (*p* < 0.001), age (*p* = 0.004) and sex (*p* = 0.024). The concentration of vitamin A increased from stage 1 to stage 5. However, the changing of vitamin A level was only correlated with eGFR staging (*p* < 0.001) and sex (*p* = 0.002). No statistical difference was noticed in vitamin B9, C or E between different stages.

**Table 4:** Serum concentrations of vitamins in CKD patients

Vitamin	CKD					*p*-value
	Stage 1 (*n* = 179)	Stage 2 (*n* = 187)	Stage 3 (*n* = 201)	Stage 4 (*n* = 97)	Stage 5 (*n* = 95)	
Vit A (µM)	0.69 (0.54, 0.92)	0.73 (0.60, 0.98)	0.85 (0.64, 1.04)	0.81 (0.67, 1.03)	1.01 (0.77, 1.34)	0.000
Vit B1 (nM)	82.91 (76.47, 91.04)	87.27 (77.65, 97.09)	86.11 (78.77, 95.79)	89.33 (80.49, 101.68)	83.81 (76.87, 92.79)	0.004
Vit B2 (nM)	11.50 (10.73, 12.36)	11.37 (10.68, 12.43)	11.29 (10.63, 12.30)	10.84 (10.26, 11.77)	11.05 (10.47, 12.01)	0.000
Vit B6 (nM)	31.30 (29.67, 33.12)	31.46 (29.44, 32.93)	31.28 (29.16, 33.21)	32.11 (29.16, 34.02)	33.67 (30.98, 34.92)	0.000
Vit B9 (nM)	20.51 (15.76, 24.88)	18.51 (14.76, 24.65)	18.2 (13.97, 23.45)	19.34 (15.41, 24.85)	19.24 (14.35, 23.87)	0.150
Vit B12 (pM)	418.5 (361.7, 448.1)	403.0 (363.0, 445.3)	390.4 (355.3, 430.8)	417.3 (381.2, 445.3)	438.2 (390.0, 464.7)	0.000
Vit C (µM)	35.44 (33.29, 38.96)	35.54 (34.04, 37.76)	35.82 (33.46, 38.38)	35.7 (34.37, 38.66)	36.36 (34.35, 38.45)	0.733
Vit D (nM)	35.68 (29.41, 42.47)	35.65 (30.03, 42.29)	35.24 (28.80, 40.78)	30.01 (24.74, 34.98)	26.12 (20.86, 30.27)	0.000
Vit E (µM)	25.72(25.03, 26.37)	25.98 (25.17, 26.58)	25.66 (24.96, 26.51)	25.56 (24.98, 26.33)	25.84 (25.10, 26.75)	0.177

Note: data were expressed as median (IQR)

**Fig. 1:**
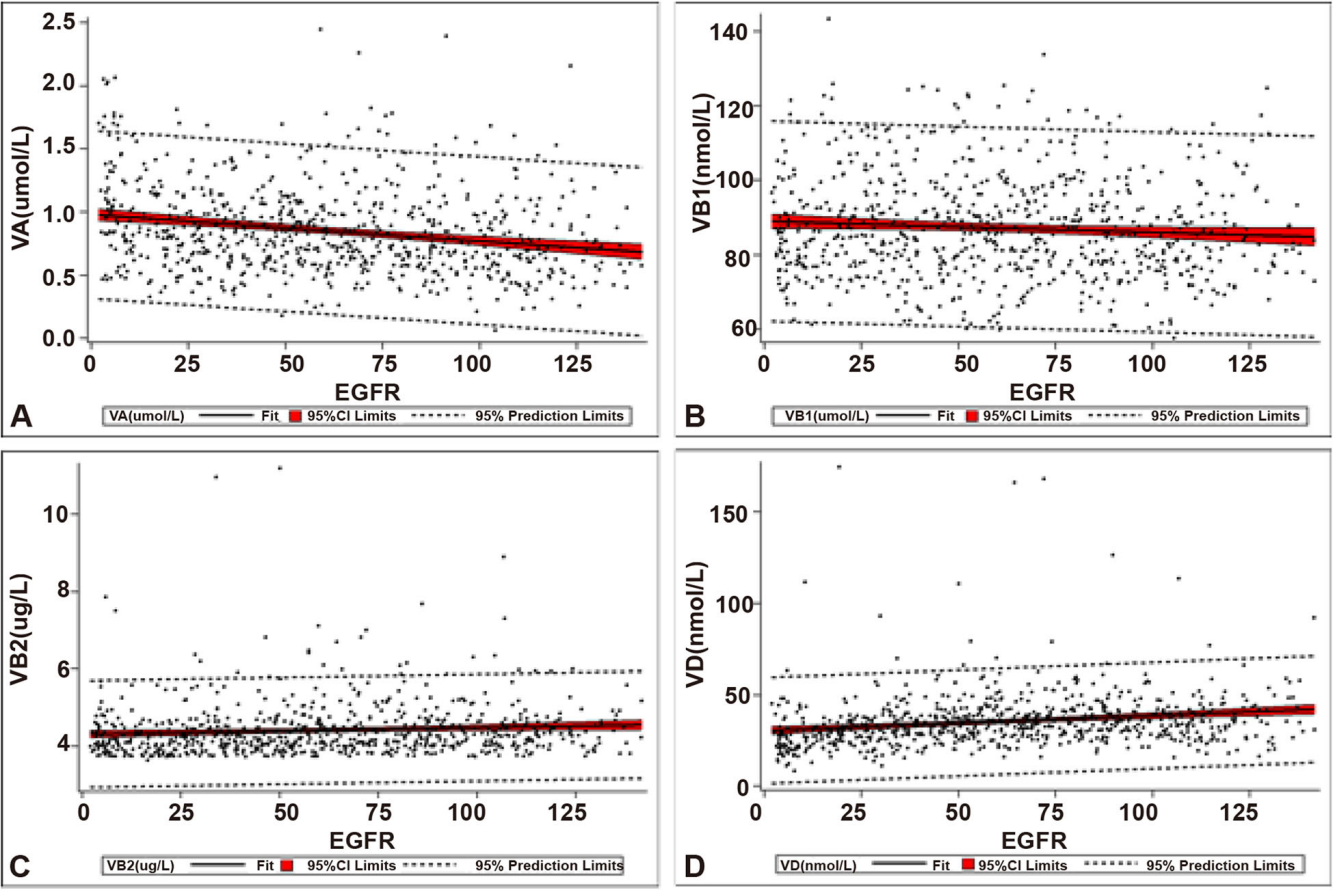
The correlation between vitamins and estimated glomerular filtration rate (eGFR). **A**, inverse correlation between vitamin A and eGFR. Pearson correlation coefficient is -0.21766 (*p* < 0.0001); **B**, inverse correlation between vitamin B1 and eGFR. Pearson correlation coefficient is -0.07844 (*p* = 0.0307); **C**, correlation between vitamin B2 and eGFR. Pearson correlation coefficient is 0.08720 (*p* = 0.0163); **D**, correlation between vitamin D and eGFR. Pearson correlation coefficient is -0.19752 (*p* < 0.0001)

No difference in vitamin levels was observed between different pathological subgroups (Table 5), suggesting no correlation between the vitamin level and pathological subgroups of CKD. Details of the correlation analysis and multiple linear regression analysis between serum levels of each vitamin and eGFR was listed in Table 6. Based on the significance tests, significant but weak correlation was found between vitamin A, B1, or D and eGFR. However, the coefficient of vitamin B1 (-0.07844, *p* = 0.0307) was too small to be practically meaningful. Vitamin A was inversely associated with eGFR (-0.21766, *p* < 0.0001), whilst vitamin D (0.19752, *p* < 0.001) was positively associated (Fig. 1).

**Table 5:** Serum concentrations of vitamins in CKD patients of different pathological subgroups

Vitamin	CKD					*p*-value
	MN	IgAN	FSGS	OPN	SN	
Vit A (µM)	0.75 (0.57, 0.96)	0.82 (0.64, 1.06)	0.78 (0.68, 0.99)	0.79 (0.61,1.04)	0.73 (0.55, 0.94)	0.063
Vit B1 (nM)	83.93 (76.82,95.16)	84.51 (75.70, 96.26)	87.23 (78.40,96.22)	83.09 (80.42,88.56)	85.59 (78.59,90.02)	0.885
Vit B2 (nM)	11.34 (10.68, 12.36)	11.42 (10.68, 12.33)	11.41 (10.63, 12.41)	11.34 (10.44, 12.22)	11.42 (10.44, 12.14)	0.912
Vit B6 (nM)	31.25 (29.37,32.73)	31.26 (28.96,32.99)	31.15 (27.95,33.18)	32.27 (29.59,34.53)	31.67 (29.94,33.63)	0.057
Vit B9 (nM)	19.81 (14.76,24.16)	19.43 (15.36,24.77)	17.90 (12.96,20.93)	19.40 (13.92,25.62)	18.28 (14.94,24.75)	0.472
Vit B12 (pM)	417.2 (364.6, 445.5)	413.3 (363.7, 448.1)	377.0 (353.6, 419.0)	408.2 (371.4, 446.5)	412.9 (354.0, 449.2)	0.260
Vit C (µM)	35.33 (33.25,38.41)	35.82 (34.00,38.46)	35.87 (33.68,37.59)	35.13 (32.69,38.81)	35.56 (34.10,37.77)	0.616
Vit D (nM)	33.56 (27.32,39.92)	35.58 (29.88, 41.45)	36.73 (28.88,43.06)	33.46 (28.19,40.51)	33.51 (27.58,39.09)	0.255
Vit E (µM)	25.91 (25.14, 26.54)	26.24 (25.07, 26.68)	25.63 (24.89, 26.26)	25.70 (24.94, 26.44)	25.61 (25.05, 26.40)	0.396

Note: data were expressed as median (IQR); *MN* membranous nephropathy; *IgAN* IgA nephropathy; *FSGS* focal segmental glomerulosclerosis; *OPN* other primary nephropathy; *SN* secondary nephropathy

**Table 6:** The correlation and regression analysis between different vitamins and estimated glomerular filtration rate (eGFR)

Variable	Pearson correlation		Multiple linear regression		
	Coefficient	*p*-value	Parameter	SE of parameter	*p*-value
Vit A	-0.21766	< 0.0001	-23.45440	3.63941	< 0.0001
Vit B1	-0.07844	0.0307	-0.20342	0.09237	0.0280
Vit B2	0.08720	0.0163	0.69456	1.98502	0.7265
Vit B6	-0.06300	0.0828	-0.12628	0.31433	0.6880
Vit B9	0.06546	0.0715	0.33561	0.21735	0.1230
Vit B12	-0.05781	0.1115	----	----	----
Vit C	-0.03097	0.3943	----	----	----
Vit D	0.19752	< 0.0001	0.46360	0.09356	< 0.0001
Vit E	0.00177	0.9611	----	----	----
Intercept	----	----	76.19669	16.60018	< 0.0001

## Discussion

CKD has become a grave public health problem worldwide, especially in developing countries. Being the largest developing country in this world, China has about 119.5 (112.9–125.0) million CKD patients [3]. The prevalence of CKD increases with age of the population [2]. In 2000, a cross-sectional study including 15,540 Chinese adults was carried out. The age-specific prevalence of CKD was 0.71 %, 1.69 %, 3.91 %, and 8.14 % among participants aged 35 to 44, 45 to 54, 55 to 64, and 65 to 74 years old, respectively [32]. In 2012, the results of a China’s national cross-sectional investigation showed that the average age of patients with CKD at stage 3–5 was 63.6 ± 14.7 [3]. In our study, the median age of CKD patients at stage 3–5 was only 50 years old, which was more than 10 years younger than in previous studies. The discrepancy in patients’ average ages may be due to the prevalence of comorbidities or the composition of pathological subgroups. For example, less than 10 % (70/759) of the patients were diagnosed with secondary nephropathy, which needs further study.

Hypertension, DM and CVD are common coexisting chronic diseases of CKD in clinical setting, where one is not necessarily more central than the other. CKD is not only a common cause of hypertension but also a complication of uncontrolled hypertension [33]. The prevalence of hypertension in patients with CKD could increase from 67 to 92 % along with the declining of eGFR in advanced stages [34]. The interaction between CKD and hypertension is complex, and is more likely to lead to poor CVD outcomes. Thus, lowing blood pressure can slow the decline of eGFR, delay the progression to end-stage renal disease (ESRD), and reduce the incidence of CVD in CKD patients as well [35]. DM is a recognized risk factor for CVD and is the leading cause of CKD. In America, the prevalence of CKD among patients with DM was about 36 %, which was more than 2 folds of general population [36]. In the present study, the prevalence of hypertension, DM, and atherosclerosis increased along with the decreasing of eGFR. For example, the prevalence of hypertension in stage 1 and stage 5 patients were 29.05 and 87.37 %, respectively. This finding was in line with previous studies [34]. In addition, the prevalence of hypertension and DM in patients with CKD at stage 3–5 was 76.1 and 26.2 %, which was much higher than a cross-sectional study reported in 2012 [3]. The higher prevalence of hypertension (76.1 % vs. 60.5 %) and DM (26.2 % vs. 19.1 %) could be one of the reasons leading to a younger median age of patients with CKD at stages 3–5 in the present study, also suggesting that patients’ ages may be on a downward trend.

Vitamins, water or fat-soluble, are important for normal cellular function as well as the growth and development of human body. Due to this, vitamin deficiency may cause severe health problem. It was reported that vitamins played important roles in the prognosis of CKD. Hyperhomocysteinemia (hHcy) was highly prevalent and strongly associated with renal function deterioration in patients with CKD. Vitamins B9 and B12 regulate the metabolism of homocysteine and reduce the odds of CKD progression by 83 % [17]. Vitamin D deficiency (VDD) had been reported to be as high as 80 % in patients with CKD, which was associated with albuminuria, faster progression of kidney disease and leading to increased all-cause mortality [13]. In this study, we investigated the status of vitamin status in 759 hospitalized patients with CKD at stage 1–5, and evaluated their association with eGFR. To our knowledge, this was the first of its kind that 9 vitamins associated with CKD were studied simultaneously. Based on the literature review, there was no consensus regarding the reference range for vitamin D deficiency. Some experts suggested serum vitamin D level should be equal or greater than 30 nmol/L, [30] while others proposed that serum level between 50 and 74 nmol/L as insufficiency, and less than 50 nmol/L as deficiency [37]. In light of the importance of vitamin D in the progression of CKD, we preferred to use a stricter reference value (50 nmol/L) to evaluate the status of vitamin D in patients with CKD. In this case, vitamin D deficiency was found in all patients of this study. A weak positive correlation (0.19752, *p* < 0.001) between vitamin D and eGFR was identified, indicating that circulating vitamin D concentrations were lower in patients of advanced stages and with lower eGFR. Vitamin D deficiency may be related to a decrease in intake from food, supplement, insufficient sunlight exposure, or generation of vitamin D3 from 7-DHC [38]. Due to the limitations of data collected in this study, it was difficult to confirm that vitamin D deficiency were attributed to the above mentioned reasons. However, it was worth noting that the urine protein of this cohort ranged from 1.23 g/d (stage 1) to 2.80 g/d (stage 5), high urinary loss of vitamin D-binding protein could be one of the reasons for vitamin D deficiency [39]. Since lower levels of vitamin D have been associated with poor outcomes of CKD, including an increased risk of progression and mortality, [11, 40] the management of vitamin D concentration should be of importance in the treatment of similar patients.

Except for vitamin D, the serum levels of other vitamins were all within the reference range or on the borderline. Circulating vitamin A and its metabolites are essential for cellular maintenance, gene regulation, lipid metabolism, and inflammatory response [41]. In the present study, an inverse relationship between vitamin A and eGFR (-0.21766, p < 0.0001) was identified, although the correlation was weak, which was in line with what was reported previously [41, 42]. Whether it was CKD or its related comorbidities that altered vitamin A homeostasis remains unknown [43–45]. To prevent toxicity, supplement of vitamin A is not recommended for CKD patients unless necessary [41]. It is notable that homocysteine level increased as the kidney function (eGFR) decreasing. In the meantime, the levels of vitamin B6, vitamin B9 and vitamin B12 didn’t show any association with Hcy, which was inconsistent with previous studies. As reported previously, plasma vitamin C concentration demonstrated a positive, linear relationship with eGFR in both diabetic and non-diabetic patients, suggesting renal dysfunction was associated with a decrease in plasma vitamin C level [14]. However, no correlation of vitamin C with eGFR was observed in this study.

The limitations to our research include (1) absence of data about dietary intake, enteral feeds, vitamin supplements, and medications related to bone and mineral metabolism of the patients, which made it difficult to test etiologic hypotheses. (2) The presence of a vitamin in serum may neither be able to reflect the adequacy and content at the tissue or cellular level nor the efficiency of utilization at the tissue or cellular level. Patients may exhibit functional deficiency of a vitamin despite normal serum content. (3) This was a single-center study conducted in a tertiary hospital. Multi-center studies with a large sample size, particularly if outpatients are included, should be carried out in the future to alleviate the possible biases and confounding factors.

## Conclusions

To our knowledge, for the first time the status of 9 vitamins were assessed in a single center study at one time. Except for vitamin D, vitamin deficiency was not a serious problem in this cohort. Weak correlation of vitamin A or D with the severity of renal function was identified. Patients’ ages have been decreasing from those in previous reports; whether it is correlated to the elevated prevalence of comorbidities, such as hypertension and/or diabetes, requires further study.

## Data Availability

The datasets used and/or analyzed during the current study are available from the corresponding author on reasonable request.

## Abbreviations

CKD: Chronic kidney disease
eGFR: Estimated glomerular filtration rate
CI: Confidence interval
hHcy: Hyperhomocysteinemia
GFR: Glomerular filtration rate
sCr: Serum creatinine
eGFR: Estimated glomerular filtration rate
CKD-EPI: CKD Epidemiology Collaboration
ALP: Alkaline phosphatase
CHOL: Total cholesterol
TG: Triglyceride
HDL: High‑density lipoprotein
LDL: Low‑density lipoprotein
Hcy: Homocysteine
DM: Diabetes mellitus
